# Supplementary material for: Syncope Time Frames for Adverse Events after Emergency Department Presentation: An Individual Patient Data Meta-Analysis
Source: Medicina (Kaunas). 2021 Nov 12;57(11):1235. doi: 10.3390/medicina57111235 (PMC8623370; doi:10.3390/medicina57111235)
Supplement: Supplementary file 1 [file medicina-57-01235-s001.zip › medicina-1418457-supplementary.pdf]

Table S1. Pooled rates of adverse outcomes at the different time frames.

	N of studies	N of events	N of patients	Pooled rate (%)	95% CI (%)	I2 (%)
**Ventricular arrhythmias**						
24 hours	8	29	11653	0.3	0.2-0.5	30
72 hours	7	33	10553	0.4	0.2-0.8	50
7-10 days	8	40	11284	0.5	0.3-0.7	47
1 month	8	47	11175	0.5	0.3-0.8	35
1 year	5	21	2917	0.9	0.6-1.4	15
**Supraventricular arrhythmias**						
24 hours	8	87	11653	0.8	0.5-1.2	48
72 hours	7	91	10553	0.9	0.7-1.2	24
7-10 days	8	94	11284	0.9	0.6-1.3	45
1 month	8	111	11175	1.1	0.8-1.5	31
1 year	5	62	2917	1.3	0.3-5.0	94
**Pause >3 seconds**						
24 hours	6	51	10593	0.4	0.2-0.7	48
72 hours	5	54	9493	0.5	0.2-1.0	52
7-10 days	6	57	10225	0.4	0.2-0.9	60
1 month	6	65	10116	0.5	0.2-0.9	64
1 year	3	17	1879	0.7	0.1-3.7	86
**Sick sinus syndrome**						
24 hours	8	67	11653	0.7	0.3-1.7	90
72 hours	7	70	10553	0.9	0.4-2.2	91
7-10 days	8	78	11284	0.9	0.4-2.0	90
1 month	8	85	11175	1.0	0.5-2.2	90

1 year	5	42	2917	1.4	0.5-3.6	88
**High grade AV block**						
24 hours	8	66	11653	0.5	0.2-1.2	87
72 hours	7	76	10553	0.8	0.4-1.8	87
7-10 days	8	82	11284	0.7	0.3-1.6	88
1 month	8	91	11175	0.9	0.5-1.8	86
1 year	5	29	2917	0.8	0.2-2.6	86
**PM or ICD malfunction**						
24 hours	8	2	11653	0.1	0.0-0.1	0
72 hours	7	3	10553	0.1	0.0-0.2	0
7-10 days	8	3	11284	0.1	0.0-0.2	0
1 month	8	4	11175	0.1	0.0-0.2	0
1 year	5	4	2917	0.3	0.1-0.7	3
**Ischemic or structural heart disease**						
24 hours	8	74	11653	0.8	0.4-1.6	83
72 hours	7	75	10553	0.9	0.4-1.9	87
7-10 days	8	105	11284	1.2	0.8-2.0	82
1 month	8	114	11175	1.3	0.8-2.2	83
1 year	5	73	2917	2.6	1.7-4.0	68
**Pulmonary embolism**						
24 hours	8	25	11653	0.3	0.2-0.4	0
72 hours	7	27	10553	0.3	0.2-0.4	0
7-10 days	8	34	11284	0.3	0.2-0.5	0
1 month	8	37	11175	0.4	0.2-0.6	18
1 year	5	14	2917	0.5	0.3-0.9	0

**Aortic dissection**						
24 hours	7	4	11282	0.1	0.0-0.0	0
72 hours	6	4	10182	0.1	0.0-0.1	0
7-10 days	7	4	10913	0.1	0.0-0.2	0
1 month	7	4	10804	0.1	0.0-0.2	0
1 year	4	2	2546	0.1	0.0-0.2	0
**Haemorrhage**						
24 hours	8	131	11653	1.3	0.8-2.2	83
72 hours	6	130	10182	1.5	0.9-2.8	86
7-10 days	7	149	10913	1.5	0.9-2.5	86
1 month	7	158	10804	1.6	1.0-2.6	86
1 year	5	51	2917	1.8	0.9-3.8	84
**Traumatic syncope recurrence**						
24 hours	5	0	9493	0.1	0.0-0.2	0
72 hours	5	2	9493	0.1	0.0-0.3	20
7-10 days	6	16	9813	0.2	0.1-0.8	75
1 month	6	24	9737	0.4	0.1-1.3	83
1 year	3	58	1350	0.9	0.0-15.6	90
**Cardiopulmonary resuscitation**						
24 hours	4	0	3827	0.1	0.0-0.3	0
72 hours	3	3	2727	0.1	0.1-0.4	0
7-10 days	5	10	4515	0.3	0.1-0.6	38
1 month	5	11	4406	0.3	0.1-0.7	53
1 year	3	9	2234	0.4	0.1-1.3	58

N: number; CI: confidence interval; PM: pacemaker; ICD: implantable cardioverter defibrillator; AV: atrioventricular.
